# Effects of Telemedicine on Informal Caregivers of Patients in Palliative Care: Systematic Review and Meta-Analysis

**DOI:** 10.2196/54244

**Published:** 2024-04-08

**Authors:** Xiaoyu Yang, Xueting Li, Shanshan Jiang, Xinying Yu

**Affiliations:** 1 Department of Oncology, Shengjing Hospital of China Medical University, Shenyang, China; 2 College of Nursing, China Medical University, Shenyang, China; 3 Department of Pediatrics, Shengjing Hospital of China Medical University, Shenyang, China

**Keywords:** telemedicine, palliative care, informal caregivers, caregiver burden, anxiety, depression, quality of life, systematic review, meta-analysis, PRISMA, Preferred Reporting Items for Systematic Reviews and Meta-Analyses

## Abstract

**Background:**

Telemedicine technology is a rapidly developing field that shows immense potential for improving medical services. In palliative care, informal caregivers assume the primary responsibility in patient care and often face challenges such as increased physical and mental stress and declining health. In such cases, telemedicine interventions can provide support and improve their health outcomes. However, research findings regarding the use of telemedicine among informal caregivers are controversial, and the efficacy of telemedicine remains unclear.

**Objective:**

This study aimed to evaluate the impacts of telemedicine on the burden, anxiety, depression, and quality of life of informal caregivers of patients in palliative care.

**Methods:**

A systematic literature search was conducted using the PubMed, Embase, Web of Science, CENTRAL, PsycINFO, CINAHL Plus with Full Text, CBM, CNKI, WanFang, and VIP databases to identify relevant randomized controlled trials published from inception to March 2023. Two authors independently screened the studies and extracted the relevant information. The methodological quality of the included studies was assessed using the Cochrane risk-of-bias tool. Intervention effects were estimated and sensitivity analysis was conducted using Review Manager 5.4, whereas 95% prediction intervals (PIs) were calculated using R (version 4.3.2) and RStudio.

**Results:**

A total of 9 randomized controlled trials were included in this study. The meta-analysis indicated that telemedicine has reduced the caregiving burden (standardized mean differences [SMD] −0.49, 95% CI −0.72 to −0.27; *P*<.001; 95% PI −0.86 to −0.13) and anxiety (SMD −0.23, 95% CI −0.40 to −0.06; *P*=.009; 95% PI −0.98 to 0.39) of informal caregivers; however, it did not affect depression (SMD −0.21, 95% CI −0.47 to 0.05; *P*=.11; 95% PI −0.94 to 0.51) or quality of life (SMD 0.35, 95% CI −0.20 to 0.89; *P*=.21; 95% PI −2.15 to 2.85).

**Conclusions:**

Although telemedicine can alleviate the caregiving burden and anxiety of informal caregivers, it does not significantly reduce depression or improve their quality of life. Further high-quality, large-sample studies are needed to validate the effects of telemedicine. Furthermore, personalized intervention programs based on theoretical foundations are required to support caregivers.

## Introduction

### Background

With the continued increase in the number of individuals with multiple and severe diseases, the global demand for palliative care services is also growing [1]. Given that most patients who require palliative care prefer to spend time at home and receive the necessary care [2-4], informal caregivers play a crucial role in caring for patients. However, the cumbersome and complex care tasks may have negative impacts on their physical, psychological, and social well-being [5-7]. In recent years, telemedicine, as an emerging technology, has been increasingly used in home care [2], benefiting informal caregivers [8,9]. It may serve as a pathway to support informal caregivers of patients in palliative care, improve their health outcomes, and thus enhance the quality of palliative care [10].

The World Health Organization estimates that 56.8 million people require palliative care yearly [11]. However, there is a prevailing shortage of professional palliative care personnel, and the majority of patients prefer to receive such care at home [2-4]. Hence, informal caregivers, usually family members or friends, assume the primary responsibility for patient care. In doing so, they adapt to changes in their role, family, and social life to provide long-term, unpaid care for patients [12,13]. Informal caregivers frequently lack professional training [4]. Thus, they face unmet supportive care needs, such as symptom management, psychological counseling, and social support [4,13], and experience anxiety, depression, physical overload, and a decline in the quality of life (QOL) [5-7]. Studies reveal that the state of informal caregivers and the condition of the patients mutually affect each other. The quality of care provided by caregivers in poor condition can be diminished, exacerbating the patient’s condition. In turn, the patient’s worsening condition can negatively affect informal caregivers [7,14,15]. Therefore, the demand to assist informal caregivers and address their physiological, psychological, and social health needs is urgent.

With the development of the information age, telemedicine has demonstrated tremendous potential in providing health care. Telemedicine refers to the use of information and communication technologies to facilitate communication between patients and health care workers for the assessment, diagnosis, treatment, and prevention of diseases, thereby improving patient health [16]. As a personalized medical approach, telemedicine overcomes the conventional care constraints of time and space; facilitates remote treatment, supervision, education, and care services; and promotes the rational distribution and refinement of medical resources [17]. Telemedicine has been widely applied in medical fields such as diabetes, chronic wounds, and cardiovascular diseases [18,19], benefiting patients and improving the health outcomes of informal caregivers [8,9].

In recent years, telemedicine has also provided novel ideas to guide palliative care [20]. In the field of palliative care, an increasing number of informal caregivers are opting to provide home care for patients who require palliative care [21]. Telemedicine facilitates real-time communication between professionals and family caregivers. This promotes information sharing; assists in the patient’s symptom management; and helps in providing health education, psychological counseling, and social support [22]. However, the outcomes of using telemedicine with informal caregivers are controversial. For example, a randomized controlled trial (RCT) by Chen et al [23] indicated that telemedicine could alleviate caregiving burden and enhance the QOL of informal caregivers. However, Dionne-Odom et al [24] found no significant difference between the telemedicine and control groups in terms of improvements in informal caregivers’ QOL, burden, or emotional state. Of the few available systematic reviews, most provide a descriptive summary of results without performing a meta-analysis to quantify the outcomes of the studies [25-27]. Thus, the intervention effects of telemedicine remain unclear.

### Objectives

Given the limitations of previous reviews, we conducted a systematic review and meta-analysis. We summarized articles on the intervention effects of telemedicine among the informal caregivers of patients in palliative care, focusing on 4 health outcomes: caregiver burden, anxiety, depression, and QOL. This provides a reference for the clinical practice of telemedicine. This is the first systematic review and meta-analysis to verify the effects of telemedicine on the outcomes for informal caregivers of patients in palliative care.

## Methods

### Overview

This systematic review adhered to the guidelines in the 2020 PRISMA (Preferred Reporting Items for Systematic Reviews and Meta-Analyses) checklist [28] (Checklist 1). In addition, it was registered on PROSPERO (CRD42023415688).

### Ethical Considerations

As all data used were obtained from previously published articles, this research did not require ethical approval from an institutional review board or informed consent from participants.

### Search Strategy

A literature search was conducted in 10 electronic databases (PubMed, Embase, Web of Science, CENTRAL, PsycINFO, CINAHL Plus with Full Text, CBM, CNKI, WanFang, and VIP) for publications dating from the establishment of each database until March 31, 2023. Following the Population, Intervention, Comparison, Outcome, and Study design principles, the searches in this systematic review were performed using Medical Subject Headings, the title or abstract, and keywords, as well as Boolean logical operations. Multimedia Appendix 1 describes the search strategy for all databases. In addition, relevant systematic reviews and references were manually screened to identify additional eligible studies.

### Study Eligibility Criteria

The inclusion criteria were as follows. (1) The study population was informal adult caregivers (aged ≥18 y) caring for patients receiving palliative care for severe diseases (eg, advanced stage, incurable “stage 4” diseases). (2) Intervention measures were being provided through the internet, applications, telephone, video, or other telemedicine technologies. (3) The control group received usual care or enhanced usual care or was on a waiting list. (4) The study reported outcomes for informal caregivers focusing on 1 or more of the following aspects: caregiver burden, anxiety, depression, or QOL. (5) The study was designed as an RCT. (6) The article was published in English or Chinese.

The exclusion criteria were as follows. (1) The publications were qualitative research, conference abstracts, letters, comments, reviews, or protocols. (2) Patients were underage (aged <18 y), or palliative care indications were unrelated to life-limiting diseases (eg, chronic diseases or nonmalignant pain). (3) Interventions were not being targeted at informal caregivers. (4) The full-text article or relevant data were not accessible.

### Study Selection and Data Extraction

The titles and abstracts of the retrieved literature were first downloaded and imported into Endnote X9 (Clarivate; a reference management program) to remove duplicates. Two evaluators then independently screened the studies based on the inclusion and exclusion criteria. Any disagreements were resolved through consultation or discussion with a third researcher. Data were extracted through a predesigned table, including the name of the first author, year of publication, country, age of the caregiver, sample size, type of disease diagnosed in the patient, type and content of intervention measures, study duration, and time of the outcome assessment.

### Quality Assessment

Two evaluators independently assessed the methodological quality of the included studies using the Cochrane Collaboration’s tool for assessing the risk of bias [29]. Seven aspects were evaluated: (1) random sequence generation, (2) allocation concealment, (3) blinding of participants and personnel, (4) blinding of outcome assessment, (5) incomplete outcome data, (6) selective reporting, and (7) other biases. Each study was categorized as “low risk,” “uncertain risk,” or “high risk,” with disagreements resolved through consultation or discussion with a third researcher.

### Data Analysis

For studies with multiple measurements, only data from the last measurement were extracted for analysis. SDs were calculated according to the *Cochrane Handbook for Systematic Reviews of Interventions* if not reported [29]. If required data were not reported, we contacted the first authors of the relevant publication. Heterogeneity testing and the meta-analysis were conducted using Review Manager 5.4 (The Cochrane Collaboration). Intervention effects were estimated through standardized mean differences (SMDs) and 95% CIs, and forest plots were generated. A 2-sided *P* value <.05 was considered statistically significant. Heterogeneity was evaluated using the *χ*^2^ test (with *P*<.10 indicating heterogeneity) and *I*^2^ test (with *I*^2^>50% indicating moderate heterogeneity and *I*^2^>75% indicating high heterogeneity). If *I*^2^≤50% and *P*>.10, a fixed-effect model was adopted for data merging and analysis; otherwise, a random-effects model was used. A sensitivity analysis was conducted using a one-study-out method to evaluate the robustness of the combined results. In addition, 95% prediction intervals (PIs) were calculated using R (version 4.3.2; R Foundation for Statistical Computing) and RStudio (Posit) to explain the heterogeneity across studies and estimate the true effects in similar future studies [30].

## Results

### Search Results and Selection

**Figure 1.**
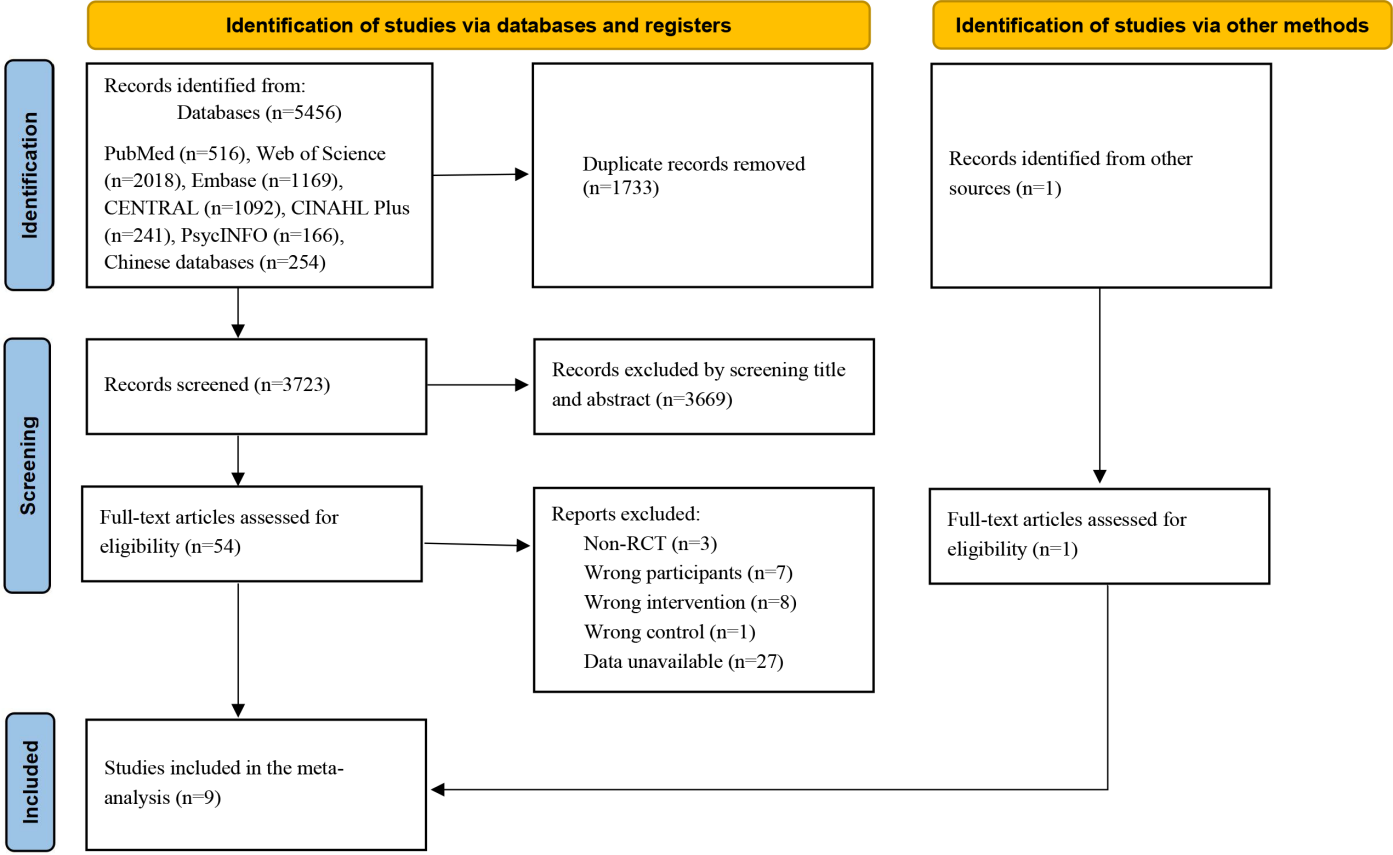
PRISMA (Preferred Reporting Items for Systematic Reviews and Meta-Analyses) flowchart. RCT: randomized controlled trial.

A preliminary search of the electronic databases yielded 5456 articles: 254 in Chinese and 5202 in English. After removing 1733 duplicated articles, an additional 3669 unrelated articles were excluded after evaluating their titles and abstracts, leaving 54 articles for the full-text review. From these, 8 articles were included, and with the addition of 1 more article, 9 studies were ultimately included in the meta-analysis. The screening process is detailed in Figure 1 [28].

### Characteristics of the Included Studies

#### Study Characteristics

Multimedia Appendix 2 [23,24,31-37] summarizes the main characteristics of the included studies. These studies were all RCTs published in 3 countries between 2015 and 2023: a total of 6 from the United States, 1 from the Netherlands, and 2 from China. Four studies mentioned the theoretical or conceptual framework of the intervention, including Erikson’s psychosocial development theory and Bowen’s family system theory [23], self-determination theory [31], shared decision-making [33], and cognitive behavioral stress management [35].

#### Characteristics of Informal Caregivers

The studies involved 1215 informal caregivers, with the number of participants in each study ranging from 35 to 334. The average age of the informal caregivers ranged from 45.71 (SD 11.85) to 60.1 (SD 12.5) years, and they were predominantly patients’ parents, spouses or partners, and children. The types of diseases of the patient included advanced cancer, advanced heart failure, and advanced dementia.

#### Characteristics of Telemedicine Interventions

Telemedicine was practiced via websites, web conferences, applications, or the telephone, but primarily through websites and the telephone. A total of 4 studies provided interventions through a website. Oliver et al [33] performed a 3-arm clinical trial, where 1 group received an intervention via Facebook, which offered education and social support to informal caregivers, whereas a separate group received the ACCESS intervention. Here, in addition to the Facebook-based intervention, web conferences were incorporated to facilitate the engagement of informal caregivers in joint decision-making in palliative care. The project aimed to alleviate informal family caregivers’ anxiety and depression. Pensak et al [35] implemented a 12-week intervention named Pep-Pal, which provided stress management support to informal caregivers of patients with advanced cancer via a website. The intervention program of Parker Oliver et al [34], ACTIVE, used web conferences or telephone calls to link informal caregivers to end-of-life care teams to improve caregivers’ perceptions of pain management. Similarly, Fu et al [37] established real-time communication between medical staff and family caregivers of patients with advanced cancer via an internet platform to provide relevant health guidance. Furthermore, 2 studies provided intervention through an application. Schuit et al [36] developed a program called Oncokompas to provide personalized information, suggestions, and supportive care solutions tailored to the caregiver’s situation. Chen et al [23] created a dyadic life review program for patients with advanced cancer and their caregivers using WeChat software to promote their QOL. In addition, 3 studies offered interventions via the telephone. Dionne-Odom et al [24] implemented a telephone intervention program named ENABLE CHF-PC, which offered psychological and problem-solving support for patients with heart failure in palliative care and their caregivers to improve their emotions, burden, and QOL. Two years later, Dionne-Odom et al [32] performed a similar intervention for patients with advanced cancer and their caregivers. Finally, Badr et al [31] provided a telephonic psychosocial intervention to enhance the QOL of patients with advanced cancer and their caregivers.

#### Characteristics of Controls

The control group in 1 study received enhanced usual care [33], whereas those in the remaining studies received usual care. Furthermore, in the study by Schuit et al [36], informal caregivers in the control group were allowed to use telemedicine equipment after the research ended.

### Risk of Bias

**Figure 2.**
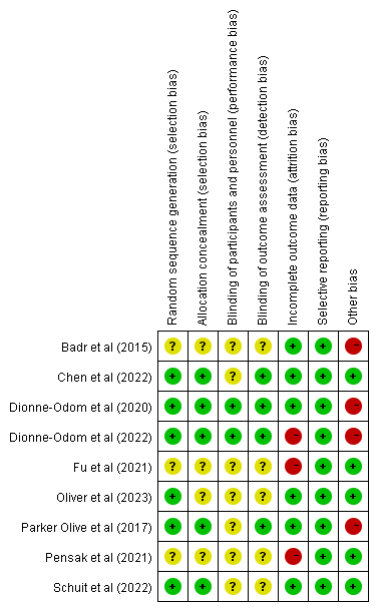
Risk of bias in each study [23,24,31-37]. Red, green, and yellow colors indicate high, low, and unclear risk of bias, respectively.

A quality assessment of the included studies was conducted using the Cochrane risk-of-bias tool. Although most studies (6/9, 67%) reported using randomization, some did not detail allocation concealment, potentially leading to selection bias. Only 2 studies were determined as having a low risk of implementation bias owing to the challenge of blinding researchers and participants in telemedicine intervention trials [24,32]. Approximately half (4/9, 45%) the studies blinded the outcome assessors, and thus, their risk of measurement bias was classified as low. Three studies were determined to have a high risk of attrition bias due to elevated loss to follow-up rates or a lack of appropriate data processing methods [32,35,37]. However, no selective reporting bias was detected in the included studies. Four studies were categorized as having a high risk of other biases due to baseline differences [24,34] and small sample sizes [31,32]. The results are shown in Figure 2.

### Meta-Analysis

#### Caregiver Burden

**Figure 3.**
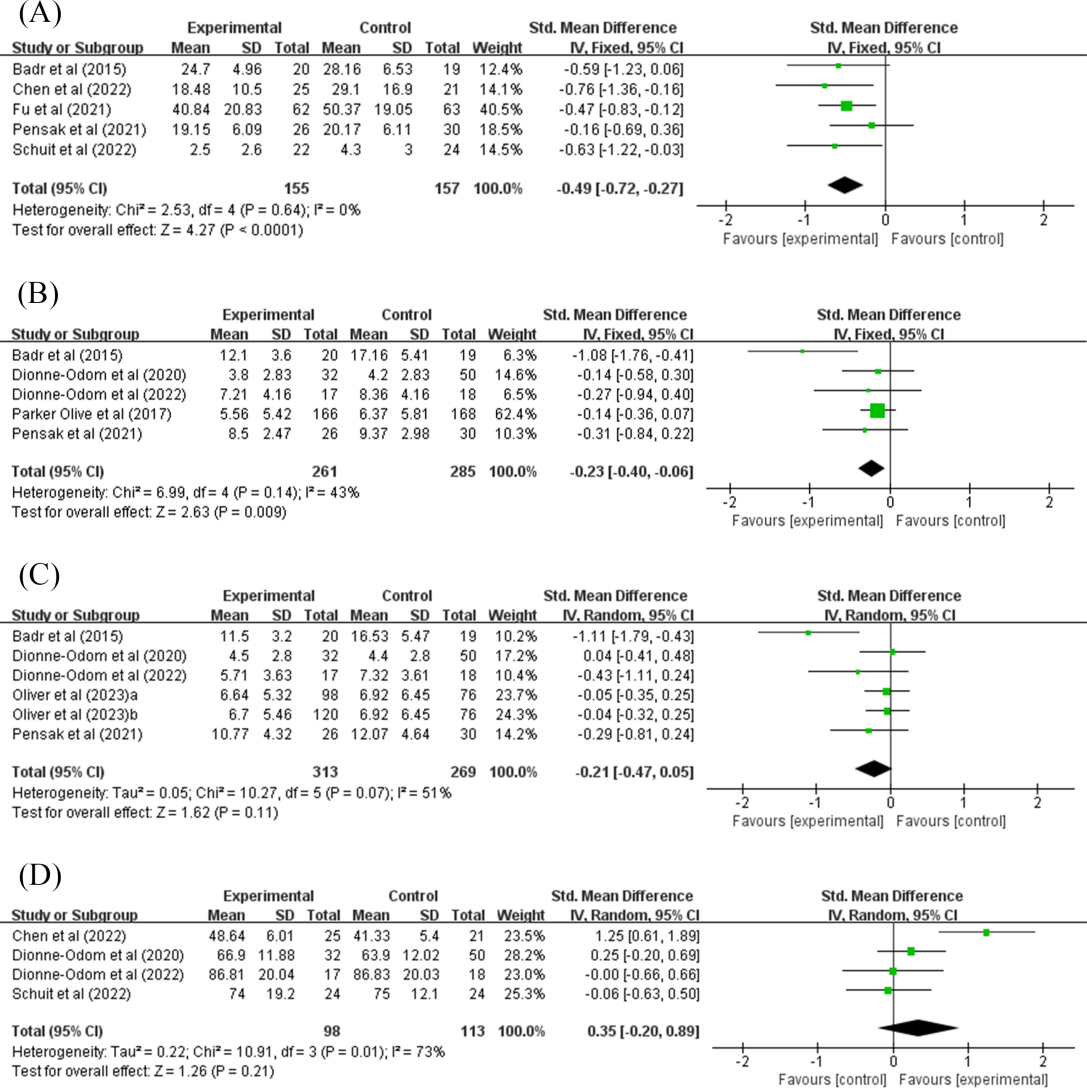
Forest plot of telemedicine versus control group: (A) caregiver burden, (B) anxiety, (C) depression, and (D) quality of life [23,24,31-37]. IV: inverse variance.

**Figure 4.**
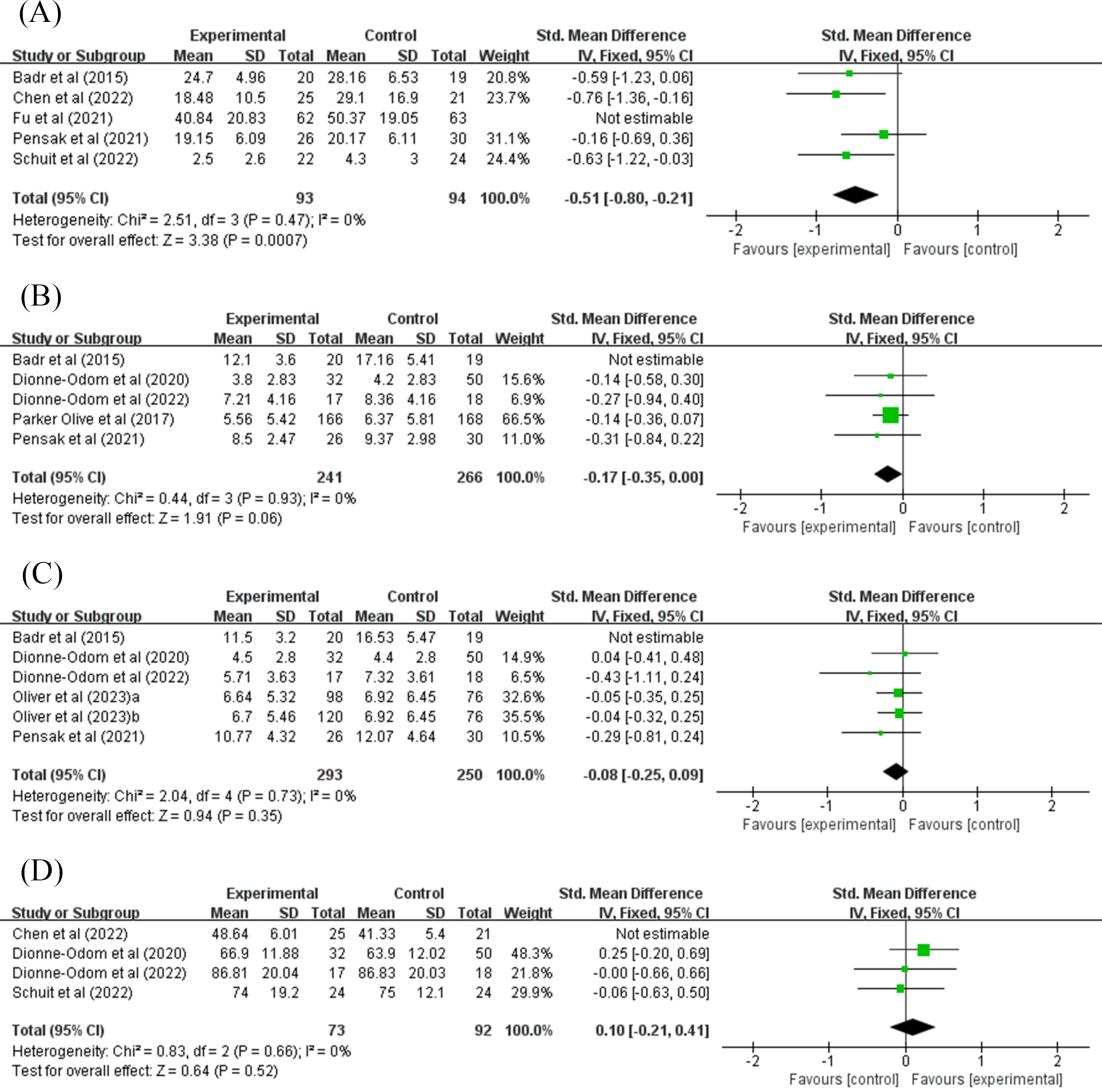
Sensitivity analyses: (A) caregiver burden, (B) anxiety, (C) depression, and (D) quality of life [23,24,31-37]. IV: inverse variance.

A total of 5 studies that evaluated caregiver burden were included in the meta-analysis [23,31,35-37]. Since no significant heterogeneity was observed among the included studies (*I*^2^=0%; *P*=.64), a fixed-effect model was used for merging the data. The results revealed that telemedicine intervention could mitigate the burden on informal caregivers (SMD −0.49, 95% CI −0.72 to −0.27; *P*<.001; 95% PI −0.86 to −0.13), as shown in Figure 3A. The sensitivity analysis showed that the results were stable, as shown in Figure 4A. The results remained unchanged when studies were merged using a random-effects model.

#### Anxiety

A total of 5 studies assessed the anxiety level of informal caregivers [24,31,32,34,35]. Due to mild heterogeneity among the included studies (*I*^2^=43%; *P*=.14), a fixed-effect model was adopted to pool the data for analysis. The results demonstrated that telemedicine intervention could reduce informal caregivers’ level of anxiety (SMD −0.23, 95% CI −0.40 to −0.06; *P*=.009; 95% PI −0.98 to 0.39), as shown in Figure 3B. The sensitivity analysis showed that after excluding Badr et al [31], the pooled results were the opposite, with *I*^2^ decreasing to 0%, as shown in Figure 4B. This could be attributed to a higher risk of bias in this study. However, when merging studies using a random-effects model, the results still retained statistical significance (SMD −0.30, 95% CI −0.57 to −0.03; *P*=.03).

#### Depression

A total of 5 studies assessed the depression level of informal caregivers [24,31-33,35]. In the study by Oliver et al [33], “a” represents the ACCESS intervention and “b” represents the Facebook intervention. Due to moderate heterogeneity among the included studies (*I*^2^=51%; *P*=.07), a random-effects model was used for merging the data. The analysis indicated that the telemedicine intervention did not result in a statistically significant difference in reducing depression among informal caregivers (SMD −0.21, 95% CI −0.47 to 0.05; *P*=.11; 95% PI −0.94 to 0.51), as shown in Figure 3C. Furthermore, the sensitivity analysis showed that no individual trial could change the results. However, after excluding Badr et al [31], the *I*^2^ decreased to 0%, as shown in Figure 4C.

#### QOL of Caregivers

A total of 4 studies that assessed QOL were included in the meta-analysis [23,24,32,36]. Due to moderate heterogeneity among the studies (*I*^2^=73%; *P*=.01), a random-effects model was used. The results indicated that the telemedicine intervention did not result in a statistically significant difference in improving the overall QOL of informal caregivers (SMD 0.35, 95% CI −0.20 to 0.89; *P*=.21; 95% PI −2.15 to 2.85), as shown in Figure 3D. Furthermore, the sensitivity analysis indicated that no individual trial could change the results. However, after excluding Chen et al [23], the *I*^2^ decreased to 0%, as shown in Figure 4D.

## Discussion

### Principal Findings

The results of this review indicate that compared to conventional care, telemedicine interventions can alleviate the caregiving burden and anxiety of informal caregivers; however, they do not significantly alleviate depression or improve their QOL. The 95% PIs indicate considerable heterogeneity among the studies, and the effects of future telemedicine interventions on these outcomes remain uncertain, except for reducing caregiver burden.

### Caregiver Burden

The results of the study demonstrated that telemedicine interventions could relieve the caregiving burden of informal caregivers, which is consistent with previous research [9,12,31]. The systematic review by Hu et al [9] demonstrates that internet-based interventions can effectively alleviate the stress of informal caregivers of patients with chronic diseases and improve their well-being. Chih et al [38] developed the Comprehensive Health Enhancement Support System for informal caregivers of patients with advanced cancer. The tool reduced the negative emotions of family caregivers and subsequently decreased their caregiving burden. Caregiver burden consists of both subjective and objective levels. Subjective burden includes the perceived physical, emotional, social, and economic difficulties caused by caring for individuals with serious diseases, whereas objective burden refers to the time and number of tasks devoted to patient care [39]. Telemedicine facilitates health education, assists in decision-making, helps develop problem-solving skills, and provides social support. It also improves and conserves the resources and time of informal caregivers. Thus, it is conducive to alleviating the caregiving burden at both the subjective and objective levels.

### Anxiety

The results revealed that telemedicine interventions can alleviate anxiety in informal caregivers, which is consistent with the findings of previous research [8,27]. Research indicates that the likelihood of anxiety occurring in caregivers of patients with advanced cancer is 3 times that of the general population [40]. Here, factors such as overwhelming nursing pressure, inadequate self-care, and the lack of supportive care can lead to anxiety [41,42]. Currently, the proposed interventions to reduce the anxiety of informal caregivers focus on psychological education, skill training, and treatment counseling [43]. In this case, telemedicine enables monitoring, assessing, and managing patient symptoms, which can enhance informal caregivers’ symptom management skills [1,37]. Moreover, it allows them to join discussions on the disease and participate in clinical decisions [20,33,44]. This can help satisfy informal caregivers’ information needs and enhance their caregiving confidence and ability. In addition, telemedicine can provide psychological interventions, improve interpersonal relationships, and offer training in stress management skills [31,32], thereby alleviating symptoms of anxiety. However, the sensitivity analysis indicated that the result was unstable. To ensure greater stability, it will be necessary to gather additional data for further investigation.

### Depression

The results of our research indicate that telemedicine does not have a statistically significant effect on alleviating depression among caregivers, which is consistent with the findings of previous research [45]. In addition to influencing factors such as the high nursing stress and insufficient social support observed for anxiety, an increased economic burden may also contribute to the onset of depression [42,46]. Despite its potential advantages, telemedicine requires a stable internet connection and available electronic devices. The initial investment cost of such equipment may negatively affect informal caregivers [47]. Furthermore, researchers only offered counseling on disease knowledge and mental and emotional well-being, but not economic and welfare support. Subsequently, factors including a lower baseline depression level in the study population [24], small sample size, and significant differences in intervention measures in various studies may negatively impact the combined results. These findings differ from those of Northouse et al [48], possibly due to variations in the target population. The study by Northouse et al [48] focused on informal caregivers of patients with cancer. In contrast, our research noted higher loss to follow-up rates in the population with advanced diseases, potentially impeding the discovery of beneficial outcomes. Moreover, Northouse et al [48] conducted a self-controlled study, whereas we included RCTs in which conventional palliative care can alleviate depression in informal caregivers [46]. Consequently, the extent to which telemedicine can improve depression is limited.

### Quality of Life

This study found that telemedicine does not significantly improve the QOL of informal caregivers of patients in palliative care, which is consistent with the findings of earlier research [25,49]. Most informal caregivers consistently place the needs of patients above their own [50], leading to various unmet supportive care needs, such as physical, psychological, and social needs [6,51], and a subsequent decline in QOL. As QOL is a multidimensional construct, a multidisciplinary intervention is often more effective than single-faceted approaches. However, the majority of studies (3/4, 75%) in this review targeted interventions at the social-psychological level, and the results might not be ideal. Furthermore, the small sample sizes in the included studies and variations in intervention measures may have limited the possibility of revealing meaningful results. Finally, the effectiveness of intervention measures may further be moderated by other factors such as the characteristics of informal caregivers, preexisting mental health issues, and the caregiver-patient relationship. Therefore, future efforts should aim to devise personalized interventions for specific informal caregiver populations to ensure the best possible support.

### Strengths and Limitations

This study meticulously adhered to the systematic review writing process, developed a comprehensive search strategy, and selected appropriate methods for meta-analysis. To enhance the reliability of the results, only RCT studies were included. However, this review also had limitations. First, we only included available data for the analysis. Missing data may impact the combined results of the meta-analysis. Second, some included studies, especially pilot studies, have small sample sizes, which requires a cautious approach to generalizing the results. Third, the inclusion of only English- and Chinese-language articles may lead to publication bias. Last, the included studies all measured the results immediately after intervention, without evaluating the persistence of the intervention effect. Therefore, in the future, large samples and high-quality research are required to further validate the intervention effects of telemedicine and explore the most suitable intervention duration for informal caregivers.

### Implications for Practice and Future Research

The findings indicated that telemedicine interventions have beneficial effects on the informal caregivers of patients in palliative care. We recommend that professional palliative care personnel consider the needs of informal caregivers, incorporating telemedicine into care plans to optimize and complement existing health care measures. When implementing such interventions, several considerations arise. First, the needs of informal caregivers are diverse and require multidisciplinary team collaboration. Second, personalized interventions should be tailored based on the demographic characteristics of informal caregivers. Finally, cost-effectiveness should be considered. In this regard, we suggest that relevant organizations establish regulations to minimize health care costs as much as possible.

Moreover, a theoretical or conceptual framework can provide the foundation of interventions, drive their development, and facilitate the prediction and explanation of their mechanism to achieve the desired effect [22]. For example, in the included studies, Badr et al [31] conducted a study based on self-determination theory. The authors hypothesized that telephone-based psychosocial interventions could improve the mental state and burden of patients and informal caregivers. Those results were statistically significant. The study by Pensak et al [35] was based on cognitive behavioral stress management theory and provided stress management training to alleviate informal caregivers’ burden. In contrast, studies lacking theoretical support failed to improve patient and informal caregiver outcomes. Therefore, it is recommended that researchers have a relevant theoretical foundation when devising intervention measures to enhance the intervention effect, which will be more likely to benefit informal caregivers.

### Conclusion

In summary, telemedicine can alleviate caregiving burden and anxiety in informal caregivers but does not significantly impact their depression and QOL. Despite certain outcomes lacking statistical significance, they retain clinical relevance for those engaged in family palliative care. We believe that support provided through telemedicine represents a viable means to ensure the continuity of care, address the needs of informal caregivers, and foster favorable outcomes. Future studies that involve large samples and high-quality research are still required to further validate the effects of telemedicine. Furthermore, intervention measures should be designed with a solid theoretical basis to the fullest extent.

## Supplementary material

10.2196/54244 Multimedia Appendix 1 Search strategy.

10.2196/54244 Multimedia Appendix 2 Characteristics of the included studies.

10.2196/54244 Checklist 1 PRISMA (Preferred Reporting Items for Systematic Reviews and Meta-Analyses) checklist.

## Abbreviations

PI: prediction interval
PRISMA: Preferred Reporting Items for Systematic Reviews and Meta-Analyses
QOL: quality of life
RCT: randomized controlled trial
SMD: standardized mean differences
