# Supplementary material for: Effects of Telemedicine on Informal Caregivers of Patients in Palliative Care: Systematic Review and Meta-Analysis
Source: JMIR Mhealth Uhealth. 2024 Apr 8;12:e54244. doi: 10.2196/54244 (PMC11024400; doi:10.2196/54244)
Supplement: Multimedia Appendix 1 [file mhealth-v12-e54244-s001.docx]

**Search strategy.**

| 1. Pubmed | |
| --- | --- |
| #1 | (telemedicine[MeSH Terms]) OR (telenursing[Title/Abstract] OR telecare[Title/Abstract] OR telecommunicat*[Title/Abstract] OR telepalliative[Title/Abstract] OR telehospice[Title/Abstract] OR "remote consult*"[Title/Abstract] OR telemonitor*[Title/Abstract] OR teleconsult*[Title/Abstract] OR telehealth*[Title/Abstract] OR app[Title/Abstract] OR apps[Title/Abstract] OR application[Title/Abstract] OR virtual medicine[Title/Abstract] OR e-health[Title/Abstract] OR m-health[Title/Abstract] OR mobile[Title/Abstract] OR cellphone[Title/Abstract] OR online[Title/Abstract] OR internet[Title/Abstract] OR website[Title/Abstract] OR smartphone[Title/Abstract] OR web-based[Title/Abstract] OR Video[Title/Abstract] OR technolog*[Title/Abstract] OR digital*[Title/Abstract] OR electronic*[Title/Abstract] ) |
| #2 | (caregivers[MeSH Terms] OR family[MeSH Terms] OR spouses[MeSH Terms]) OR (caregiver[Title/Abstract] OR caregivers[Title/Abstract] OR carer[Title/Abstract] OR carers[Title/Abstract] OR family[Title/Abstract] OR families[Title/Abstract] OR spouse[Title/Abstract] OR spouses[Title/Abstract] OR "care giver"[Title/Abstract] OR "care givers"[Title/Abstract] OR relative[Title/Abstract] OR relatives[Title/Abstract] OR partner[Title/Abstract] OR partners[Title/Abstract]) |
| #3 | palliative care[MeSH Terms] OR palliative medicine[MeSH Terms] OR hospice care[MeSH Terms] OR hospices[MeSH Terms] OR terminal care[MeSH Terms] |
| #4 | "supportive care"[Title/Abstract] OR palliative[Title/Abstract] OR hospice[Title/Abstract] hospices[Title/Abstract] OR advanced[Title/Abstract] OR end-of-life[Title/Abstract] OR end-stage[Title/Abstract] OR terminal[Title/Abstract] OR incurable[Title/Abstract] |
| #5 | #3 OR #4 |
| #6 | ("Randomized Controlled Trial" [Publication Type]) OR (RCT[Title/Abstract] OR RCTs[Title/Abstract] OR randomized[Title/Abstract] OR randomly[Title/Abstract]) |
| #7 | #1 AND #2 AND #5 AND #6 |
| #8 | review[Title] |
| #9 | #7 NOT #8 |
| 1. CENTRAL | |
| #1 | MeSH descriptor: [Telemedicine] explode all trees |
| #2 | MeSH descriptor: [Telenursing] explode all trees |
| #3 | MeSH descriptor: [Telecommunications] explode all trees |
| #4 | #1 OR #2 OR #3 |
| #5 | (telemedicine OR telenursing OR telecare OR telecommunicat* OR telepalliative OR telehospice OR “remote consult*” OR telemonitor* OR teleconsult* OR telehealth* OR app OR apps OR application OR virtual medicine OR e-health OR m-health OR mobile OR cellphone OR online OR internet OR website OR smartphone OR web-based OR Video OR technolog* OR digital* OR electronic*):ti,ab,kw |
| #6 | #4 OR #5 |
| #7 | MeSH descriptor: [Caregivers] explode all trees |
| #8 | MeSH descriptor: [Spouses] explode all trees |
| #9 | (caregiver OR caregivers OR carer OR carers OR family OR families OR spouse OR spouses OR "care giver" OR "care givers" OR relative OR relatives OR partner OR partners):ti,ab,kw |
| #10 | #7 OR #8 OR #9 |
| #11 | MeSH descriptor: [Palliative Care] explode all trees |
| #12 | MeSH descriptor: [Terminal Care] explode all trees |
| #13 | MeSH descriptor: [Hospices] explode all trees |
| #14 | MeSH descriptor: [Hospice Care] explode all trees |
| #15 | ("supportive care" OR palliative OR hospice OR hospices OR advanced OR end-of-life OR end-stage OR terminal OR incurable):ti,ab,kw |
| #16 | #11 OR #12 OR #13 OR #14 OR #15 |
| #17 | MeSH descriptor: [Randomized Controlled Trial] explode all trees |
| #18 | ("Randomized Controlled Trial" OR RCT OR RCTs OR randomized OR randomly):ti,ab,kw |
| #19 | #17 OR #18 |
| #20 | #6 AND #10 AND #16 AND #19 |
| 1. Web of Science | |
| #1 | TS=(caregiver OR caregivers OR carer OR carers OR family OR families OR spouse OR spouses OR "care giver" OR "care givers" OR relative OR relatives OR partner OR partners) |
| #2 | TS=("supportive care" OR palliative OR hospice OR hospices OR advanced OR end-of-life OR end-stage OR terminal OR incurable) |
| #3 | TS=(telemedicine OR telenursing OR telecare OR telecommunicat* OR telepalliative OR telehospice OR "remote consult*" OR telemonitor* OR teleconsult* OR telehealth* OR app OR apps OR application OR virtual medicine OR e-health OR m-health OR mobile OR cellphone OR online OR internet OR website OR smartphone OR web-based OR Video OR technolog* OR digital* OR electronic*) |
| #4 | TS=("Randomized Controlled Trial" OR RCT OR RCTs OR randomized OR randomly) |
| #5 | #1 AND #2 AND #3 AND #4 |
| #6 | TI=(review) |
| #7 | #5 NOT #6 |
| 1. Embase | |
| #1 | 'telemedicine'/exp OR 'telehealth'/exp OR 'telecare'/exp |
| #2 | telemedicine:ti,ab,kw OR telenursing:ti,ab,kw OR telecare:ti,ab,kw OR telecommunicat*:ti,ab,kw OR telepalliative:ti,ab,kw OR telehospice:ti,ab,kw OR 'remote consult*':ti,ab,kw OR telemonitor*;ti,ab,kw OR teleconsult*:ti,ab,kw OR telehealth*:ti,ab,kw OR app:ti,ab,kw OR apps:ti,ab,kw OR application:ti,ab,kw OR virtual medicine'ti,ab,kw OR 'e-health':ti,ab,kw OR 'm-health':ti,ab,kw OR mobile:ti,ab,kw OR cellphone:ti,ab,kw OR online:ti,ab,kw OR internet:ti,ab,kw OR websiteti,ab,kw OR smartphone:ti,ab,kw OR 'web-based':ti,ab,kw OR video OR technolog*:ti,ab,kw OR digital*:ti,ab,kw OR electronic*:ti,ab,kw:ti,ab,kw |
| #3 | #1 OR #2 |
| #4 | 'caregiver'/exp OR 'spouse'/exp |
| #5 | caregiver:ti,ab,kw OR caregivers:ti,ab,kw OR carer:ti,ab,kw OR carers:ti,ab,kw OR family:ti,ab,kw OR families:ti,ab,kw OR spouse:ti,ab,kw OR spouses:ti,ab,kw OR 'care giver':ti,ab,kw OR 'care givers':ti,ab,kw OR relative:ti,ab,kw OR relatives:ti,ab,kw OR partner:ti,ab,kw OR partners:ti,ab,kw |
| #6 | #4 OR #5 |
| #7 | 'palliative therapy'/exp OR 'hospice care'/exp OR hospice/exp OR 'terminal care'/exp |
| #8 | 'supportive care':ti,ab,kw OR palliative:ti,ab,kw OR hospice:ti,ab,kw OR hospices:ti,ab,kw OR advanced:ti,ab,kw OR 'end-of-life':ti,ab,kw OR'end-stage':ti,ab,kw OR terminal:ti,ab,kw OR incurable:ti,ab,kw |
| #9 | #7 OR #8 |
| #10 | 'randomized controlled trial'/exp |
| #11 | 'randomized controlled trial':ti,ab,kw OR rct:ti,ab,kw OR rcts:ti,ab,kw OR randomized:ti,ab,kw OR randomly:ti,ab,kw |
| #12 | #10 OR #11 |
| #13 | #3 AND #6 AND #9 AND #12 |
| #14 | review:ti |
| #15 | #13 NOT #14 |
| 1. PsycINFO | |
| S1 | (DE "Telemedicine") |
| S2 | TI (telemedicine OR telenursing OR telecare OR telecommunicat* OR telepalliative OR telehospice OR “remote consult*” OR telemonitor* OR teleconsult* OR telehealth* OR app OR apps OR application OR virtual medicine OR e-health OR m-health OR mobile OR cellphone OR online OR internet OR website OR smartphone OR web-based OR Video OR technolog* OR digital* OR electronic*) OR AB (telemedicine OR telenursing OR telecare OR telecommunicat* OR telepalliative OR telehospice OR “remote consult*” OR telemonitor* OR teleconsult* OR telehealth* OR app OR apps OR application OR virtual medicine OR e-health OR m-health OR mobile OR cellphone OR online OR internet OR website OR smartphone OR web-based OR Video OR technolog* OR digital* OR electronic*) |
| S3 | S1 OR S2 |
| S4 | (DE "Caregivers") OR (DE "Spouses") |
| S5 | TI(caregiver OR caregivers OR carer OR carers OR family OR families OR spouse OR spouses OR "care giver" OR "care givers" OR relative OR relatives OR partner OR partners) OR AB (caregiver OR caregivers OR carer OR carers OR family OR families OR spouse OR spouses OR "care giver" OR "care givers" OR relative OR relatives OR partner OR partners) |
| S6 | S4 OR S5 |
| S7 | (DE "Palliative Care") OR (DE "Hospices") OR (DE "Terminal Care") |
| S8 | TI("supportive care" OR palliative OR hospice OR hospices OR advanced OR end-of-life OR end-stage OR terminal OR incurable) OR AB("supportive care" OR palliative OR hospice OR hospices OR advanced OR end-of-life OR end-stage OR terminal OR incurable) |
| S9 | S7 OR S8 |
| S10 | (DE "Randomized Controlled Trials") |
| S11 | TI("Randomized Controlled Trial" OR RCT OR RCTs OR randomized OR randomly) OR AB ("Randomized Controlled Trial" OR RCT OR RCTs OR randomized OR randomly) |
| S12 | S10 OR S11 |
| S13 | S3 AND S6 AND S9 AND S12 |
| 1. CINAHL | |
| S1 | (MH "Telehealth+") OR (MH "Telemedicine+") |
| S2 | TI (telemedicine OR telenursing OR telecare OR telecommunicat* OR telepalliative OR telehospice OR “remote consult*” OR telemonitor* OR teleconsult* OR telehealth* OR app OR apps OR application OR virtual medicine OR e-health OR m-health OR mobile OR cellphone OR online OR internet OR website OR smartphone OR web-based OR Video OR technolog* OR digital* OR electronic*) OR AB (telemedicine OR telenursing OR telecare OR telecommunicat* OR telepalliative OR telehospice OR “remote consult*” OR telemonitor* OR teleconsult* OR telehealth* OR app OR apps OR application OR virtual medicine OR e-health OR m-health OR mobile OR cellphone OR online OR internet OR website OR smartphone OR web-based OR Video OR technolog* OR digital* OR electronic*) |
| S3 | S1 OR S2 |
| S4 | (MH"Caregivers") OR (MH"Spouses") |
| S5 | TI(caregiver OR caregivers OR carer OR carers OR family OR families OR spouse OR spouses OR "care giver" OR "care givers" OR relative OR relatives OR partner OR partners) OR AB (caregiver OR caregivers OR carer OR carers OR family OR families OR spouse OR spouses OR "care giver" OR "care givers" OR relative OR relatives OR partner OR partners) |
| S6 | S4 OR S5 |
| S7 | (MH "Palliative Care") OR (MH "Palliative Medicine") OR (MH "Hospice Care") OR (MH "Hospices") OR (MH "Terminal Care") OR (MH "Hospice and Palliative Nursing") |
| S8 | TI("supportive care" OR palliative OR hospice OR hospices OR advanced OR end-of-life OR end-stage OR terminal OR incurable) OR AB("supportive care" OR palliative OR hospice OR hospices OR advanced OR end-of-life OR end-stage OR terminal OR incurable) |
| S9 | S7 OR S8 |
| S10 | (MH "Randomized Controlled Trials+") |
| S11 | TI("Randomized Controlled Trial" OR RCT OR RCTs OR randomized OR randomly) OR AB ("Randomized Controlled Trial" OR RCT OR RCTs OR randomized OR randomly) |
| S12 | S10 OR S11 |
| S13 | S3 AND S6 AND S9 AND S12 |
| 1. CNKI/VIP/CBM/WangFang | |
| #1 | 主题=(远程 OR 信息化系统 OR 移动程序 OR 手机 OR 电话 OR APP OR 网络 OR 互联网 OR 智能 OR 智慧 OR 电子 OR 移动 OR 微信 OR 虚拟医疗) |
| #2 | 主题=(姑息 OR 临终 OR 晚期 OR 末期) |
| #3 | 主题=(照顾 OR 照护 OR 家庭 OR 配偶 OR 伴侣 OR 家属 OR家人) |
| #4 | #1 AND #2 AND #3 |
