# Supplementary material for: Effects of Telemedicine on Informal Caregivers of Patients in Palliative Care: Systematic Review and Meta-Analysis
Source: JMIR Mhealth Uhealth. 2024 Apr 8;12:e54244. doi: 10.2196/54244 (PMC11024400; doi:10.2196/54244)
Supplement: Multimedia Appendix 2 [file mhealth-v12-e54244-s002.docx]

**The characteristics of included studies.**

| **Study** | **Country** | **Participants** | | | | **Type of patient** | **Intervention** | | | **Control** | **Time**  **(weeks)** | **Outcome** | |
| --- | --- | --- | --- | --- | --- | --- | --- | --- | --- | --- | --- | --- | --- |
|  |  | **Relationship**  **to patient** | **Age**  **I/C** | **Sample Size** | |  | **Type** | **Content** | **Format** |  |  | **scale** | **Assessment time** |
|  |  |  |  | **I** | **C** |  |  |  |  |  |  |  |  |
| Chen  2022[23] | China | Spouse, Child,  Parent | 50.9(13.8)/  45.7 (11.8) | 25 | 21 | Advanced cancer | Psychosocial support | Review, evaluate,  and integrate life experiences | APP | usual care | 4 | QOLLTI-F,  ZBI | post- intervention |
| Dionne-Odom  2020[24] | USA | Spouse/partner, Parent, Other | 58.2(12.4)/  57.6 (10.8) | 82 | 76 | Advanced Heart Failure | Psychosocial and problem-solving support | Provide education, nurture, advice,  and comprehensive heart failure care | telephone | usual failure care | 48 | BCOS, HADS, MBCB | 8 and 16 weeks |
| Badr  2015[31] | USA | Spouse/partner, Child, Other | 51.1(10.2)^c^ | 20 | 19 | Advanced cancer | Psychosocial support | Teach skills, support autonomy, improve interpersonal connections or the sense of relatedness | telephone | usual care | 6.5 | PROMIS, ZBI | post- intervention |
| Dionne-Odom  2022[32] | USA | Spouse/partner, Parent, Other | 55.5(14.2)/  55.5(13.7) | 17 | 18 | Advanced cancer | Comprehensive support | Review skills in stress management, self-care, getting help, staying organized, and future planning | telephone | usual care | 24 | CQOL-C, HADS, | 8 weeks and  post- intervention |
| Oliver  2023[33]^a^ | USA | Spouse/partner, Child, Other | 56.5(12.0)/ 57.8 (12.2) | 98 | 76 | Hospice patients with cancer | Information and emotional support | Provide educational and social support to caregivers, and involve them in sharing decision-making | Website | enhanc-ed usual care | 13 | PHQ-9 | 14, 30, 60, and 90 days |
| Oliver  2023[33]^b^ | USA | Spouse/partner, Child, other | 55.0(12.8)/  57.8(12.2) | 120 | 76 | Hospice patients with cancer | Information and emotional support | Provide educational and social support | Website | enhanc-ed usual care | 13 | PHQ-9 | 14, 30, 60 and 90 days |
| Parker Oliver  2017[34] | USA | Spouse, Child,  Other | 60.1(12.5)/  59.2 (13.3) | 166 | 168 | Cancer, dementia, other hospice patients | Information support | Caregivers attended care planning meetings and provided feedback | Website or  telephone | usual hospice care | 4 years | CQLI-R,  GAD-7 | Once every 2 weeks for 4 weeks, then once a month for 6 months, and every 45 days thereafter |
| Pensak  2021[35] | USA | Spouse/partner,Other | 53.3(14.7)/  55.1 (10.9) | 26 | 30 | Advanced cancer | Psychosocial support | Provide stress management training | Website | usual care | 12 | HADS, CES–D,  PSS | post- intervention |
| Schuit 2022[36] | Nether-lands | Partner | 57.0(10.0)/  58.0 (13.0) | 24 | 28 | Advanced cancer | Comprehensive support | Provide information, advice, and supportive care options tailored to the caregiver's situation | APP | usual care+  waitlist | 13 | EQ-5D VAS,  CSI | 2 weeks and post- intervention |
| Fu  2021[37] | China | Parent, Child, Other | 48.0(12.0)/  50.6 (13.5) | 62 | 63 | Advanced cancer | Comprehensive support | Knowledge and skills education, information exchange, real-time communication, symptom monitoring, and health guidance | Website | usual care | 13 | CBS-CP | post- intervention |

Note: a, ACCESS intervention; b, Facebook intervention; c, combined intervention and control data;

Abbreviations: I=Intervention, C=Control; CQOL-C=the 35-item Caregiver Quality of Life-Cancer; EQ-5D VAS=the visual analog scale of the self-report questionnaire EuroQol-5D; BCOS= the 15-item Bakas Caregiving Outcomes Scale; CQLI-R=the Caregiver Quality of Life Index-Revised; QOLLTI-F=the Quality of life in Life-threatening Illness-Family Carer Questionnaire; HADS=The 14-item Hospital Anxiety and Depression Scale; PROMIS=the Six Patient-Reported Outcome Measurement Information System Measures Short Form; GAD-7=the Generalized Anxiety Disorder scale; CES-D=the center of Epidemiologic Studies Depression scale; PHQ-9=the Patient Health Questionnaire-9; ZBI=the 12-item Zarit Caregiving Burden Scale; CBS-CP=Caregiver Burden Scale for Cancer Patients; PSS=Perceived Stress Scale; CSI=the Caregiver Strain Index; MBCB=the 14-item Montgomery-Borgatta Caregiving Burden Scale.
